# Bergamot (*Citrus bergamia*): A Potential New Nutraceutical Against Cellular and Physiological Alterations Induced by Emerging Contaminants in Sentinel Organisms

**DOI:** 10.3390/antiox14050539

**Published:** 2025-04-30

**Authors:** Federica Impellitteri, Cristiana Roberta Multisanti, Kristian Riolo, Giorgia Zicarelli, Miriam Porretti, Giovanna Cafeo, Marina Russo, Paola Dugo, Giuseppa Di Bella, Giuseppe Piccione, Alessia Giannetto, Caterina Faggio

**Affiliations:** 1 Department of Veterinary Sciences, University of Messina, Viale Giovanni Palatucci SNC, 98168 Messina, Italy; federica.impellitteri@studenti.unime.it (F.I.); cristiana.multisanti@studenti.unime.it (C.R.M.); gpiccione@unime.it (G.P.); 2 Department of Chemical, Biological, Pharmaceutical and Environmental Sciences, University of Messina, Viale Ferdinando Stagno d’Alcontres, 31, 98166 Messina, Italy; kristian.riolo@unime.it (K.R.); giorgyz@live.it (G.Z.); 3 Auribondo APL Swift Services (Malta) Ltd., HF26, Hal Far Industrial Estate, BBG 3000 Birzebbugia, Malta; miriam.porretti@studenti.unime.it; 4 Messina Institute of Technology c/o Department of Chemical, Biological, Pharmaceutical and Environmental Sciences, University of Messina, Viale Giovanni Palatucci SNC, 98168 Messina, Italy; giovanna.cafeo@studenti.unime.it (G.C.); marina.russo@unime.it (M.R.); paola.dugo@unime.it (P.D.); 5 Dental, Morphological and Functional Images Sciences, Department of Biomedical, University of Messina, (BIOMORF), 98100 Messina, Italy; giuseppa.dibella@unime.it; 6 Department of Eco-Sustainable Marine Biotechnology, Stazione Zoologica Anton Dohrn, 80100 Naples, Italy

**Keywords:** *Citrus bergamia*, nutraceuticals, emerging contaminants, cytotoxicity, gene expression, aquatic environment

## Abstract

Nutraceuticals are gaining research interest due to their beneficial potential and their use to counter the impact of emerging contaminants on natural ecosystems. Particularly, during the COVID-19 pandemic, the use of personal hygiene/care products and disinfectants increased significantly. These products contain several substances in their formulations, including surfactants, which have proven to be hazardous to the entire aquatic ecosystem. In the present study, bergamot (*Citrus bergamia*) peel extract was used as a nutraceutical to counteract the toxicity of sodium lauryl sulphate (SLS), a common anionic detergent with antimicrobial activity. Specimens of *Mytilus galloprovincialis*, were exposed to SLS (0.01 mg/L), bergamot peels’ extract (BRG: 5 mg/L), and their mixture for 14 days. The cellular and physiological alterations in haemocytes, digestive gland (DG) and gill cells were analysed. The analyses included cell viability of haemocytes and DG cells (trypan blue exclusion assay and the neutral red retention test); the ability of DG cells to regulate their volume (RVD); haemocyte phagocytic activity; expression of genes involved in antioxidant response (*Cu/ZnSOD, MnSOD, Hsp70*, and *CYP4Y*) on gills and DG; the energy efficiency of the organism through byssus production; and the measurement of key macromolecules, including total lipid and fatty acid content, total protein, tocopherols and carotenoids, which play a key role in maintaining physiological and metabolic functions in the organism. Overall, significant differences emerged between the control (CTR) and treated groups, with the CTR and BRG groups resembling each other, while the SLS-treated groups showed significant alterations. Meanwhile, the groups exposed to the combination showed a recovery, suggesting the potential beneficial effect of the BRG.

## 1. Introduction

The potential of natural-derived compounds in mitigating the adverse effects of emerging contaminants is receiving particular interest from the scientific community. In this context, nutraceutical compounds have shown promising biological activities in improving physiological functions and promoting general health. These include a wide range of natural bioactive compounds derived from plants, marine organisms and microorganisms, resulting in a very effective and high bioavailable resource. Indeed, their potential as sustainable solutions to pollutant-induced toxicity offers innovative approaches to address the pressing need for effective and environmentally friendly mitigation strategies [1,2,3,4] to evaluate the effectiveness of these methods in mitigating the adverse effects of emerging contaminants on aquatic organisms [5].

This study selected bergamot (*Citrus bergamia*) as the nutraceutical to be analysed. Almost all citrus species originate from the subtropical or tropical regions of Asia and Malaysia; their arrival in Italy dates back to the 17th century. This plant has a wide distribution from the Mediterranean to South America. The Mediterranean diet, defined as part of the “Intangible Cultural Heritage of Humanity”, is associated with improved health, particularly in terms of reducing the incidence of cancer, diabetes mellitus and, more importantly, coronary heart disease in Italy [6]. In particular, reducing the consumption of red meat and increasing the consumption of olive oil and plant-based foods has a positive effect on the onset and progression of heart disease [6]. In this context, bergamot has also attracted the attention of the perfume and pharmaceutical industry due to its chemical composition, which includes several bioactive essential oils such as limonene, a monoterpene, linalool, linalyl acetate, γ-terpinene and β-pinene [7]. It is also rich in flavonoid glycosides such as neoeriocitrin, neohesperidine, naringin, and glycosylated polyphenols such as bruteridine and melitidine [8]. Essential oils extracted from *C. bergamia* have been used as remedies for various ailments, such as parasitic infections, sore throat, and wound healing [9,10,11].

This study investigates the potential of bergamot to mitigate or prevent damage induced by SLS in aquatic model organisms. Since the onset of the COVID-19 pandemic, the use of detergents, disinfectants and foaming agents in healthcare and industry has increased dramatically. This increase is due to the need to maintain high standards of hygiene and to control microbial growth, reflecting the increased efforts to reduce the risk of infection [12,13]. Hygiene and personal care products contain several substances in their formulations, including surfactants. These molecules are essential components of many household cleaners due to their surface-active properties. They are also widely used in various industrial processes, household cleaning, and in the production of pesticides, and are increasingly recognised as emerging contaminants [14]. Among the most known surfactants, sodium lauryl sulphate (SLS) is an important example [15]. SLS is an anionic detergent known for its antimicrobial properties. It is widely used as a synthetic detergent in various household cleaning products, including laundry, and dishwasher detergents. Freitas et al. [16] indicated that SLS can enter the environment mainly through the land application of sewage sludge, the discharge of wastewater from sewage treatment plants, and the release of industrial waste into rivers and coastal areas. Although SLS biodegrades from 45% to 95% within 24 h, its continuous release into the environment leads to persistently high levels of contamination in aquatic systems [17]. In this study perspective, studies by Freitas et al. [18,19,20] identified the toxic effects of sodium lauryl sulphate on *Mytilus galloprovincialis*.

This study investigates the potential beneficial and protective effects of bergamot juice extracts from the “*Fantastico*” cultivar on the Mediterranean mussel (*M. galloprovincialis*) exposed to SLS. The mussel was chosen as a model organism because of its biologically relevant properties [21,22,23]. The effects were assessed by examining cellular and physiological changes in haemolymph, digestive gland (DG), and gill cells. In particular, haemocytes play a key role in the body’s initial immune response to xenobiotics and pathogens. The DG functions as a primary detoxification site, making it a critical target organ for contaminants. The gills, on the other hand, represent one of the first points of contact between the organism and the environment, as they are directly exposed during the filtration process, which provides the organism with nutrients and oxygen.

Furthermore, the energy yield of the organism through byssus production was quantified and qualitatively analysed. Finally, specific analyses were carried out to assess key macromolecules, including total lipid and fatty acid content, total protein, tocopherols, and carotenoids, which play a crucial role in maintaining the physiological and metabolic functions of organisms, as well as being a source of essential components in the diet (such as essential fatty acids). Macronutrients, such as lipids and proteins, are involved in many vital functions of aquatic organisms. They have already been shown to be useful trophic markers for tracking predator–prey relationships and defining food webs in the marine environment [24,25]. In addition, lipids and fatty acids are essential for energy storage, membrane structure, and signalling. At the same time, proteins are the basic building blocks of cell structures and enzymes that support growth and repair. Tocopherols and carotenoids are essential antioxidants that protect cells from oxidative stress.

The novelty of this study lies in the potential increase in knowledge on the beneficial effects of compounds of natural origin, adding these data, conducted using mussels as a model organism, to those reported by a series of recent studies that have demonstrated the beneficial potential of these substances on various fish species [26,27,28]. Therefore, the aim of this study is to assess how the co-presence of a natural compound may or may not restore the physiological parameters and nutritional profile of mussels exposed to the stress imposed by emerging contaminants. In particular, SLS was selected as it represents a highly utilized molecule despite its proven toxicity, and our hypothesis of using BRG as a mitigating agent could be exploited as a basis for proposing the inclusion of this compound in SLS-based products. Furthermore, this study lays the foundation for proposing the use of such substances to mitigate the impact of common environmental contaminants on organisms.

## 2. Materials and Methods

### 2.1. Experimental Design

During the experiment, 200 specimens of *Mytilus galloprovincialis*, all approximately with the same size, were used. The average weight of the viscera was 4.45 ± 1.37 g, while the shell length measured 5.23 ± 0.55 cm and the shell width was 2.48 ± 0.23 cm. The specimens were sourced from a commercial farm at Lago di Faro, provided by the company FARAU SRL, Frutti di Mare of Messina, Italy. The mussels were sub-divided into four groups (with two replicates of 25 individuals per group) and acclimatised for approximately two weeks in eight 25 L aquariums, each equipped with oxygen aerators. The mussels were provided with filtered, nutrient-enriched lake water from the same company that supplied us the animals. The water quality parameters were salinity 3.4 ± 0.05%, pH 7.5 ± 0.01, and temperature 18.32 ± 0.11 °C, all of which were monitored after each water change. For the welfare of the animals and to maintain the quality, water in the tanks was changed every two days. Moreover, to maintain the natural animals’ circadian rhythm, experimental room was illuminated by natural light.

The exposure concentrations in the groups were as follows: SLS (0.010 mg/L), Bergamot (BRG, 5 mg/L), and the mixture (MIX, 0.010 mg/L SLS + 5 mg/L BRG), administered for a 14-day period. Additionally, the untreated control groups (CTR) were included. Stock solutions of SLS, BRG, and MIX were prepared at the specified concentrations. The water in each aquarium was changed three times a week, and chemical concentrations were maintained throughout the experiment. No mortality was recorded during the exposure period. After the acclimatisation period, substance concentrations were restored after each water change.

### 2.2. Chemical Characterization of Bergamot Extract

#### 2.2.1. Materials

The acetonitrile, water, formic acid, ethanol, and methanol were acquired from Merck Life Science (Merck KGaA, Darmstadt, Germany). The eriocitrin, ferulic acid, narirutin, neohesperidin, and synapic acid were obtained from Extrasynthese (Genay Cedex, France). The apigenin 6,8-di-C-glucoside, diosmetin 6,8-di-C-glucoside, naringin, brutieridin, limonin glucoside, nomilin glucoside, nomilinic acid glucoside, limonin, melitidin, nomilin, and neoeriocitrin were previously isolated in our laboratory [10,29,30,31].

#### 2.2.2. Samples and Bioactive Molecules Extraction

The bergamot (*Citrus bergamia*) peels analysed belong to Castagnaro cultivar. The fruits were collected at the same stage of ripeness in Calabria (Melito di Porto Salvo, Reggio Calabria, Italy) in December 2023. The fruits were peeled, and the peels obtained were dried at 25 °C for 48 h. Before the HPLC analysis, the bergamot peels were subjected to a solvent extraction procedure, previously validated by Russo et al. [10]. Then, 10 g of peel powder (obtained from grinding dried bergamot peels) were extracted with 100 mL of methanol for 10 min using an ultrasonic bath. This procedure was repeated three times. The three aliquots were combined, filtered on filter paper, and brought to dryness in a rotary evaporator. Finally, the extract was solubilised in 10 mL of a mixture of water/ethanol (8:2, *v*/*v*), diluted 100 times with water, and injected into the HPLC systemin triplicate.

#### 2.2.3. Chromatographic Instrumentation and Method

For the RP-HPLC/PDA/MS analyses, a Shimadzu Prominence LC-20A system coupled with a photodiode array detector (SPD-M20A UV) and a single quadrupole mass spectrometer (HPLCMS-2020) was employed (Shimadzu, Milan, Italy). The analytical method employed was previously developed and validated by Russo et al. [31]. Briefly, the separation was achieved using an Ascentis Express C18 column (150 × 4.6 mm, 2.7 μm) (Merck KGaA, Darmstadt, Germany) as the stationary phase, water/formic acid (99.9:0.1, *v*/*v*) as the mobile phase A, and acetonitrile/formic acid (99.9:0.1, *v*/*v*) as the mobile phase B. Pumps were set in gradient mode at a flow-rate of 0.7 mL/min. The gradient program was as follows: 0 min, 5% B, 40 min, 25% B, 60 min, 100% B. The injection volume was 2 μL. The photodiode array detector was employed in the range 190–400 nm and the chromatograms were extracted at 280 and 325 nm. The time constant and sample frequency were 0.64 s and 1.5625 Hz, respectively. The MS acquisition was performed using an electrospray ionization source (ESI), operated in negative ionization mode. The total flow was split to waste (500 μL) and to interface (200 μL) by means of a flow splitter. MS parameters were set as follows: mass spectral range, 100–700 m/z; scan speed, 938 amu/s; interval, 0.5 s; nebulizing gas flow, 1.5 L/min; ESI temperature, 350 °C; heat block, 300 °C; desolvation line (DL) temperature, 300 °C; DL voltage, 34 V; probe voltage, +4.5 kV; Qarray voltage, 1.0 V; and detection gain, 1.05 kV. To quantify flavonoids, limonoids, and phenolic acids in the bergamot peel extract, calibration curves were constructed for each standard, in the 0.2–100 mg/L concentration range (five concentration levels, five replicates for each level) according to the PDA signal. The MS signals were employed only for the identification of bioactive compounds. The analytical method was validated according to the EURACHEM guidelines.

### 2.3. Animal Samples Collection

After the exposure, for the nutritional profile analysis, *n* = 10 organisms were randomly selected from each tank of the experimental group, and their whole body was dissected, pooled, freeze-dried, grounded into a powder, and stored at −80 °C until the analyses were performed. Total lipid, protein, FA composition, tocopherol, and carotenoids were assessed as well. In order to perform the conversion of data from dry weight to wet weight, the moisture content of each mussel pool (approximately 1 g) was evaluated. In order to identify the number and the length of byssal plates, four mussel specimens were randomly selected from each experimental group. For the purposes of the biological analysis, four organisms were randomly selected from each experimental group and pooled. The cell viability and the ability to regulate volume decrease and phagocytosis were assessed. The haemolymph was collected from the anterior adductor muscle of mussels from each experimental and control group using a glass syringe with a 23-gauge needle, in accordance with the methodology described by Bolognesi and Fenech [32]. Following the collection of the haemolymph, each mussel pool was then sacrificed on ice, and the digestive glands (DG) were dissected and harvested.

### 2.4. Byssus Analysis

Following the period of acclimatisation, the byssus was cut with surgical scissors as close to the shell as possible. The specimens were then transferred individually into glass petri dishes and placed in the respective tanks, with two specimens per tank. Following a 14-day exposure period, the byssus was carefully detached from the mussel and the length and number of individual plates were quantified using an AxioCam MRc 5 (Carl Zeiss MicroImaging, Inc., Wetzlar, Germany) camera with images captured using AxioVision AC Rel. 4.5 software.

### 2.5. Total Lipid and Fatty Acid Composition

Lipid extraction from pooled mussel samples was performed following the guidelines of the Organisation for Economic Co-operation and Development (OECD, 2012, https://mneguidelines.oecd.org/mneguidelines/, accessed on 26 March 2025). Freeze-dried and powdered samples (~4 g each) were extracted according to the method described by Porretti et al. [33]. The organic phase containing fatty acid methyl esters (FAMEs) was analysed using a gas chromatograph (GC) equipped with a split/splitless injector and a flame ionisation detector (FID) (Dani Master GC1000, Dani Instrument, Milan, Italy) in accordance with the instrument condition described previously by Porretti et al. [33]. Nutritionally significant FAMEs were identified by comparison with the retention times of a commercial reference standard mixture (FAMEs reference standards C4–C24, Supelco, Bellefonte, PA, USA). The relative percentage of each FAME was calculated based on the total chromatogram area from triplicate analyses.

### 2.6. Total Proteins

Crude protein content was determined using the Kjeldahl method, as outlined in our previous study on marine organisms [33]. Approximately 1 g of powdered sample, consisting of pooled mussels, was analysed. The crude protein on a dry weight (dw) basis for each sample was calculated based on the nitrogen content obtained from the assay, following the formula:

(1) Protein (%, dw)=% nitrogen×6.251

protein content was then expressed as a percentage of fw. All samples were analysed in triplicate, along with the analytical blanks, and the results were reported based on the converted fw.

### 2.7. Carotenoids Content

To analyse and quantify the carotenoids, expressed as β-carotene, we adapted a method by Rotondo et al. [34] using 0.5 g of lyophilised samples from each group. An amount of 10 mL of n-hexane is added, extracted by ultrasound water bath for 10 min, and the mixture was centrifuge for 10 min at 4000 rpm at 25 °C. The supernatant was separated, and the solid phase was re-extracted with the same amount of n-hexane. The supernatant phases were combined and dried by a Rotavapor (Büchi V700, BUCHI Labortechnik AG, Flawil, Switzerland). After the evaporation, samples were dissolved into 2 mL of methanol and methyl tert-butyl ether (MTBE) in 1:1 ratio.

### 2.8. Tocopherols Content

The total lipid fraction was extracted using the Folch method, and tocopherol analysis was conducted following the protocol outlined by Lo Turco et al. [35]. The analysis was carried out with a high-performance liquid chromatography system coupled to a fluorescence detector (HPLC-FD, prominence HPLC system with RF-20A detector, Shimadzu), in accordance with the conditions previously described by Lo Turco et al. [35]. Reference retention times for commercial standards of α-, γ-, and δ-tocopherol were used for the identification. Six-point calibration curves for each tocopherol were employed for quantification using the external calibration method. The total tocopherol content was determined as the sum of the individual tocopherols. All analyses were performed in triplicate.

### 2.9. Cell Viability of Haemocytes and Isolated Digestive Gland Cells

The impact of SLS, bergamot and their combination on the viability of haemolymph and DG cells was investigated using a colourimetric assay with trypan blue (TB, Sigma-Aldrich) exclusion. The assay was conducted on cells extracted from pooled mussels and evaluated in accordance with the methodology described. TB is a dye that selectively permeates non-viable cells with a compromised membrane since, under physiological conditions, viable cells resist the dye. The cells are stained with a 1:1 ratio of haemolymph and DG cells to dye for a period of five minutes. The proportion of cells that had not been stained by TB in relation to the total number of cells present in the suspension was indicative of the extent of cellular damage. This was calculated using the following formula:

(2) Cells viability %=number of viable cellstotal number of cells×100

Following the incubation period, the cells were observed under a microscope (Carl Zeiss Axioskop 20, Wetzlar, Germany) for up to 10 min, with a Burker chamber at a magnification of 40×. This was deemed an appropriate timeframe for monitoring the behaviour of the dye, given that prolonged exposure may result in it crossing the barrier of a healthy membrane.

### 2.10. Regulatory Volume Decrease Assay

The Regulatory Volume Decrease (RVD) assay was performed by placing a drop of DG cell samples on a polylysine-coated slide to promote cell adhesion. Observations were made using a Carl Zeiss Axioskop 20 light microscope (Wetzlar, Germany) equipped with a Canon 550D digital camera at 100× magnification. Samples were first rinsed with an isotonic solution (1100 mOsm) containing 550 mM NaCl, 12.5 mM KCl, 8 mM MgSO_4_, 4 mM CaCl_2_, 10 mM glucose, 40 mM MgCl_2_ and 20 mM HEPES. Three images were taken sequentially. The slide was then washed with a hypotonic solution (800 mOsm) containing 350 mM NaCl, 12.5 mM KCl, 8 mM MgSO_4_, 4 mM CaCl_2_, 10 mM glucose, 40 mM MgCl_2_, and 20 mM HEPES. A total of 15 cells per experimental condition were selected for analysis and their areas were measured using ImageJ software, version 1.54i (NIH, Bethesda, MD, USA), allowing a comparative assessment of cell areas between the control and treated groups.

### 2.11. RNA Extraction and cDNA Synthesis

Gills and DG were collected from treated and untreated mussels and used for total RNA extraction via the TRI Reagent^®^ RNA/DNA/Protein Isolation Reagent (ZYMO RESEARCH) as described by Impellitteri et al. [36] The extracted RNA was assessed for quantity and quality by using a NanoDrop 2000 spectrophotometer (Thermo Fisher Scientific, Monza, Italy) and 1% (*w*/*v*) agarose gel electrophoresis. The cDNA synthesis was performed using 1 µg total RNA and the ImProm-II™ Reverse Transcription System kit (Promega), as detailed by Riolo et al. [37].

### 2.12. Quantitative Gene Expression Analysis via qPCR

The obtained cDNA samples were first diluted (1:20) before running the quantitative polymerase chain reactions to evaluate the mRNA levels of the genes associated with the antioxidant and cytoprotective responses, namely superoxide dismutases (MnSOD and Cu/ZnSOD), cytochrome P450 (CYP4Y1), and heat shock protein 70 (Hsp70). Quantitative PCR runs were performed using the GoTaq^®^ qPCR Master Mix kit (Promega) and specific primers [37] in a Rotor-Gene Q2 plex Hrm thermocycler (Qiagen). For each qPCR reaction biological samples (*n* =6) from each experimental group were run in duplicate. No template and reverse transcriptase negative controls were run in all the reactions. Among the reference genes, the most stable (ef1- α) was used to normalize the expression levels of target genes using the ΔΔCt method.

### 2.13. Statistical Analysis

Data were subjected to statistical analysis using the SigmaPlot software (Systat software, https://grafiti.com/sigmaplot-detail/ accessed on 26 March 2025). The normality and homogeneity of the data were checked using the Kolmogorov-Smirnov and Levene tests after the one-way analysis of variance (ANOVA) and Student-Newman-Keuls post-hoc tests were used to evaluate differences in viability, RVD and the gene expression levels among groups (*p* < 0.05).

## 3. Results

### 3.1. Chemical Characterization of Bioactive Molecules in Bergamot Extract

**Figure 1:**
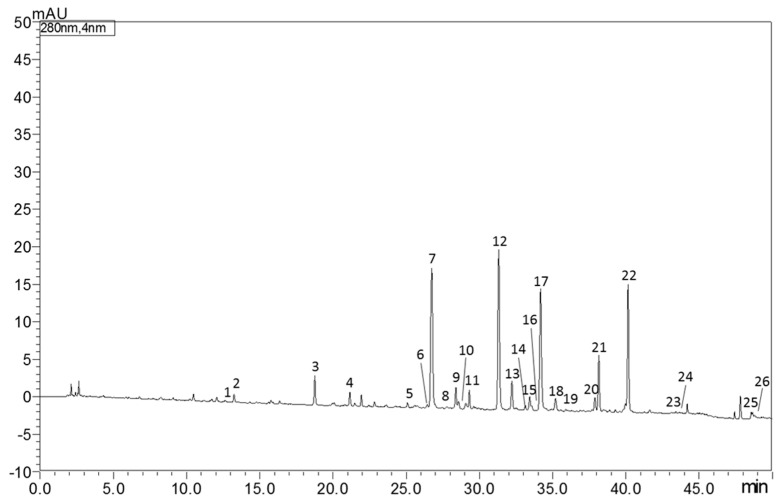
HPLC-PDA chromatogram of bioactive molecules in “*Fantastico*” bergamot peels extract at 280 nm. For peak identification see Table 1.

The identification and quantification of bioactive molecules in Castagnaro bergamot peels extract were carried out using the RP-HPLC/PDA/MS instrument. As reported in Table 1, 26 compounds, between flavonoids (F), phenolic acids (PA) and limonoids (L), were quantified. According to previously published results, neohesperidin (12,907.6 mg Kg^−1^) was the most abundant flavonoid characterized, followed by poncirin (1803.9 mg Kg^−1^) and narirutin (1673.6 mg Kg^−1^). Figure 1 shows the RP-HPLC/PDA chromatogram of bioactive molecules in “*Fantastico”* bergamot peels extracted at 280 nm.

### 3.2. Byssus Alteration

**Figure 2:**
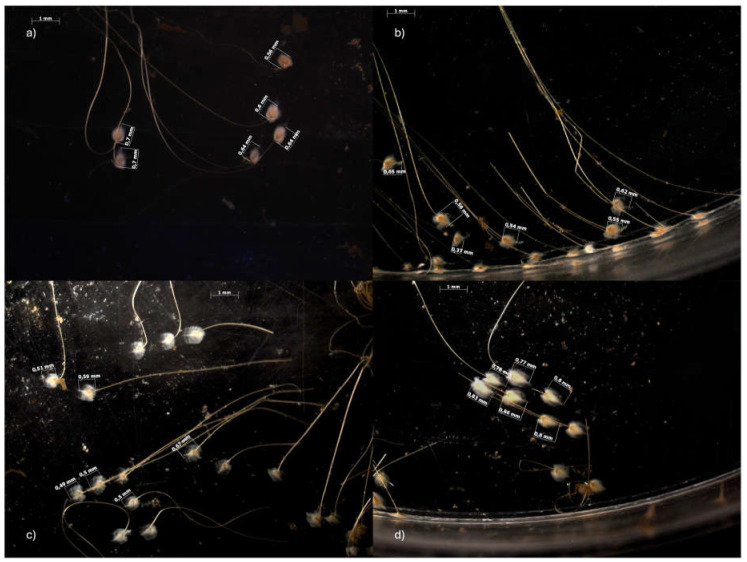
Representative images of byssus mussels taken using the AxioCam MRc 5 stereomicroscope and the AxioVision AC Rel. 4.5 software. The images show the changes in plate length from the control group (**a**) to the SLS (**b**), BRG (**c**) and MIX (**d**) exposure groups after 14 days of exposure.

Exposure to SLS, bergamot and their mixture resulted in a change in the number of abyssal plates and their length. Table 2 and Figure 2 show the average number of plaques and the observed changes in plate length following the 14-day exposure. It can be observed that plaques number in the SLS-treated group (60.75 ± 19.31) is significantly lower than that of the control (102.75 ± 14.93), with a further significant reduction in the MIX group (38.00 ± 29.47). In contrast, bergamot did not affect number of plaques (101.75 ± 30.18). The results of the static analysis indicated that the averages between the control and the mix, and of bergamot with the mix, were significantly different (*p* < 0.05). A significant reduction in the mean length can be observed compared to the control group (0.75 ± 0.14). In fact, in the SLS and BRG groups, the mean length was 0.61 ± 0.12 and 0.61 ± 0.11, while in the MIX group, it was 0.65 ± 0.14. The statistical analysis revealed a statistically significant difference between the control and all experimental groups (*p* < 0.05).

### 3.3. Total Lipid and Fatty Acid

The total content lipid and the (fatty acid) FA profile obtained from the four test pools are reported in Table 3. The control group exhibited a total lipid content of 2.21% fw, whereas the SLS-exposed group demonstrated a decline, with 1.43% fw. In the group that underwent exposure to bergamot extract, a total lipid concentration of 2.06% fw was observed. In contrast, the MIX group, exposed to both SLS and BRG, displayed values of 1.93% fw. The statistical analysis demonstrated that there were no statistically significant differences between the SLS, BRG and MIX experimental groups and the control group (*p* > 0.05). The analysis of the fatty acid profile shows that exposure to the different compounds and their mixture resulted in a significant variation in content. Regardless of the control or experimental status, the FA composition of *M. galloprovincialis* was characterised by a high content of polyunsaturated fatty acids (PUFA, 42.96–49.76%, *p* < 0.05), followed by an intermediate level of saturated fatty acids (SFA, 29.67–32.80%, *p* < 0.05) and a lower amount of monounsaturated fatty acids (MUFA, 19.33–23.14%, *p < 0.05*). The most abundant fatty acids in mussel soft tissue were eicosapentenoic acid (C20:5 *ω*-3, 20.25–20.96%, *p* > 0.05), palmitic acid (C16:0, 15.97–20.02%, *p* < 0.05), docosahexaenoic acid (C22:6 *ω*-3, 11.51–17.35%, *p* < 0.05), stearic acid (C18:0, 5.15–8.77%, *p* < 0.05), and palmitoleic acid (C16:1 *ω*-7, 0.16–8.64%, *p* < 0.05) (Table 3).The exposure to SLS very often negatively affected the lipid metabolism of *M. galloprovincialis*, both compared to the control and to the groups exposed to bergamot or MIX. On the contrary, very often the group exposed to bergamot extract showed no significant differences compared to the control, only in the case of the sum of monounsaturated fatty acids, where even the group exposed to bergamot showed a higher content (CTR: 19.33%, BRG: 23.14%), mainly due to a higher contribution of palmitoleic acid (C16:1 *ω*-9, CTR: 0.10%, BRG: 11.07%), vaccenic acid (C18:1 *ω*-7, CTR: 2.64%, BRG: 3.54%), and gondonic acid (C20:1 *ω*-9, CTR: 0.61%, BRG: 2.54%). In the group exposed to sodium lauryl sulphate, there was a general decrease in the content of saturated fatty acids (from SLS: 29.67% to 31.88–32.80% in CTR, BRG and MIX), mainly due to a significant decrease in palmitic acid (C16:0, 15.97–20.02%, *p* < 0.05). The group exposed to the mixture of the two substances often showed values similar to those of the control or bergamot, as in the case of saturated fatty acids (SFA, CTR: 32.80%, BRG: 31.88%, MIX: 32.68%, *p* > 0.05).

### 3.4. Total Protein, Carotenoids and Tocopherols Contents

The total protein content of the *M. galloprovincialis* pools from the control and exposure groups is shown in Table 4. The average protein content in the control mussels was 20.21% and decreased in all exposure groups, in SLS it was 18.16%, in BRG 18.41% and in MIX 18.57%. Static analysis showed that the difference between the sample means of all experimental groups was not large enough to be statistically significant (*p* ≥ 0.05).

The carotenoid content, expressed as β-carotene of the control and exposure groups is shown in Table 4. The mean carotenoid content in the control group was 5.99 mg/Kg, with a decrease in each exposure group. In the SLS-exposed group, there was an average content of 3.70 mg/Kg, in BRG 4.75 mg/Kg, and MIX had the highest average value of 7.44 mg/Kg. There was only a significant difference between the group exposed to SLS and the one exposed to MIX (*p* < 0.05).

The tocopherol expressed as vitamin E of the control and exposure groups is shown in Table 4. The mean vitamin E content did not vary significantly between the groups exposed to the various substances and the control (*p* > 0.05). As CTR: 0.46 mg/Kg, SLS: 0.40 mg/Kg, BRG: 0.35 mg/Kg and MIX: 0.48 mg/Kg.

### 3.5. Cell Viability

Tests conducted using TB staining demonstrated that exposure to SLS resulted in a significant decline in cell viability in both haemocytes and hepatocytes (Table 5). Haemocytes treated with SLS exhibited a statistically significant reduction in viability (93.54%), The results demonstrated a statistically significant reduction in cell viability (*p* < 0.01) in both the SLS-treated and BRG-treated groups (97.22%, *p* < 0.05; 96.00%, *p* < 0.05) in comparison to the control. In contrast, DG cells did not exhibit a significant alteration in viability, although a slight reduction was observed in the SLS-treated group (97.83%).

### 3.6. Regulatory Volume Decrease Assay

**Figure 3:**
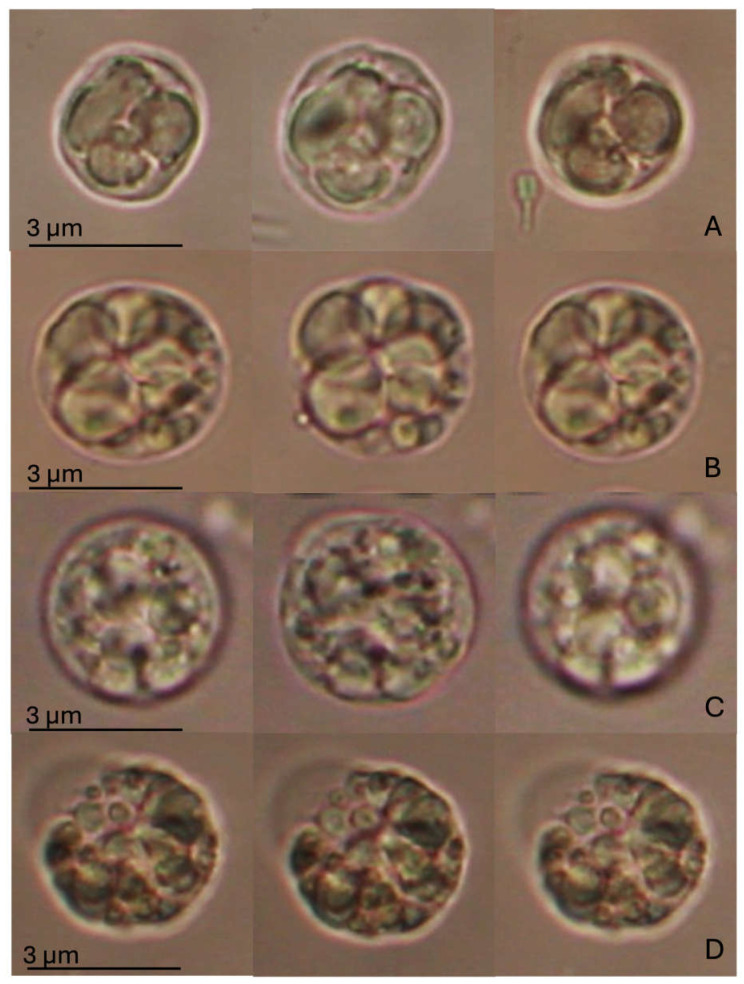
RVD assay in digestive gland cells of *Mytilus galloprovincialis* exposed to different experimental groups (SLS: 0.01 mg/L, BRG: 5 mg/L, and their combination: 0.01 + 5 mg/L) for 14 days, compared to the Control. The figure displays representative images of a single digestive gland cell observed under an isotonic solution. The three images represent crucial moments of the RVD assay, i.e., control condition (time 0); after 1 min in a hypotonic solution; after 40 min in a hypotonic solution. (**A**) Control group; (**B**) SLS; (**C**) BRG; (**D**) Mix (SLS + BRG). No significant differences were observed between the control and experimental groups or among the experimental groups.

The Regulatory Volume Decrease (RVD) assay did not indicate any statistically significant alteration, as shown in Figure 3. However, changes in cell swelling capacity, followed by recovery of the natural volume, were observed between cells exposed to SLS and MIX compared to the CTR and BRG experimental groups. *M. galloprovincialis* DG cells in the control group showed an increase in volume of about a quarter of their initial volume during exposure to the hypotonic solution, followed by a return to their original size at the end of the hypotonic wash (after 40 min). A similar trend was observed for cells exposed to BRG, which reached a swelling comparable to that of the control and then fully recovered their original volume. In contrast, cells exposed to SLS and MIX showed intermediate swelling responses compared to those of the control and BRG groups. Moreover, at the end of washing with hypotonic solution, they failed to fully recover their original volume. In particular, the SLS-treated cells increased their volume by approximately one-fifth of their original size, whereas the MIX-treated cells reached maximum swelling slightly earlier, with a smaller change in volume than the SLS group. In contrast to the CTR and BRG groups, both SLS- and MIX-treated cells showed a volume reduction from their initial size at the end of the RVD process. Notably, this reduction was more pronounced in the SLS-exposed cells, whereas the MIX-treated cells showed a relatively smaller volume reduction.

### 3.7. Antioxidant and Cytoprotective Responses

**Figure 4:**
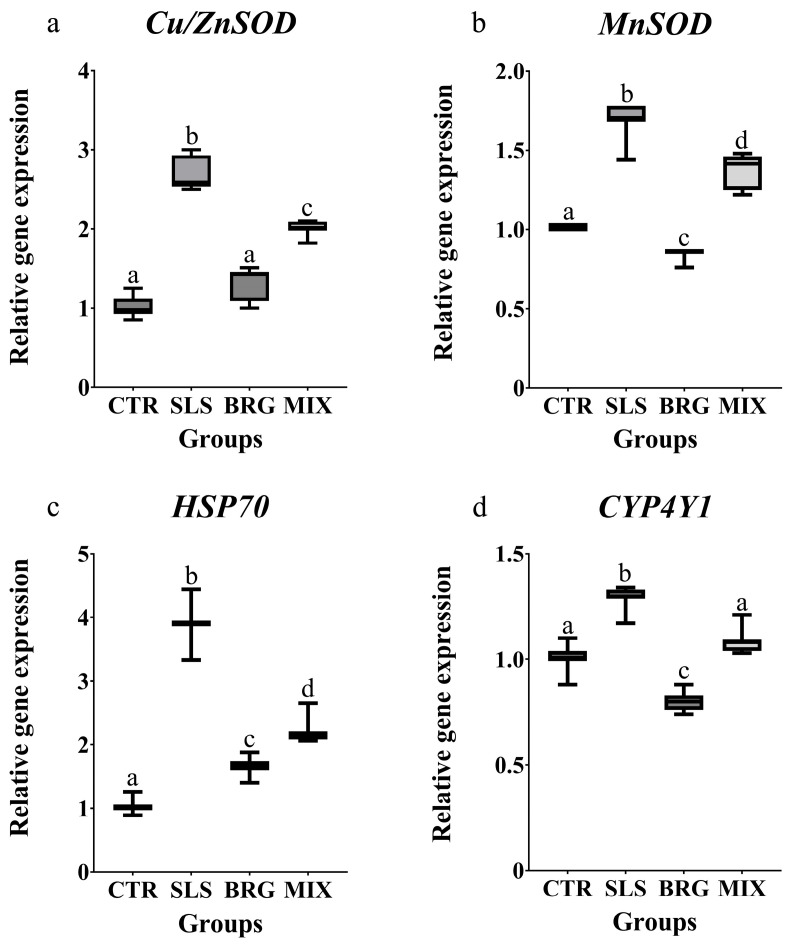
Relative gene expression of (**a**) *Cu/ZnSOD*, (**b**) *MnSOD*, (**c**) *HSP70*, and (**d**) *CYP4Y1* evaluated in gills of mussels exposed for 14 days to 0.01 mg/L of SLS (SLS), 5 mg/L of BRG (BRG) and their combination of 0.01 mg/L SLS + 5 mg/L BRG (MIX). The results are shown as mean ± SD (n = 6) and different letters represent significant differences (*p* < 0.05) among the different groups.

The transcript levels of genes associated with antioxidant and cytoprotective responses were modulated similarly in all the experimental groups. The treatment with SLS induced a significant upregulation (*p* < 0.05) of all the investigated genes (Cu/ZnSOD, MnSOD, Hsp70 and CYP4Y1) when compared to the control group (Figure 4). On the contrary, the treatment with BRG extract alone did not modulate the Cu/ZnSOD transcript levels (Figure 4a), that were comparable to the control group while the MnSOD and CYP4Y1 (Figure 4b,c) transcript levels were reduced by 1.13-fold and 1.25-fold with respect to the control group, respectively. The combined exposure to both SLS and BRG extracts (MIX group) decreased all the gene expression levels in respect to the SLS group.

## 4. Discussion

SLS is a surfactant widely distributed in the aquatic environment due to its high production volumes and numerous applications in various fields. A significant number of studies have demonstrated the adverse effects of SLS throughout the trophic chain, including humans, resulting in adverse toxicity. In this context, it is crucial to research compounds that can potentially safeguard aquatic life and to suggest the use of substances capable of mitigating the impact of environmental contaminants on organisms.

The present study demonstrates that bergamot extract, a fruit with a distinctive greenish-yellow colour and sour taste, can mitigate the adverse effects of SLS exposure on *M. galloprovincialis.* SLS is rarely monitored and remains unregulated because it is considered environmentally friendly, despite its biodegradable nature [38]. Nevertheless, recent studies have assessed the toxicity of SLS to mussels [16,17,18,19,20] and other aquatic species [36]. Macronutrients, including lipids and proteins, play a crucial role in the functioning of organisms in the aquatic environment. These compounds are useful as trophic markers to track predator–prey relationships and define food webs in the marine environment. This is because some of them, such as essential fatty acids, can only be gained from specific food resources [24,25]. In addition, macronutrients are considered to be highly effective biomarkers for the assessment of the health of ecosystems and as indicators of stress in response to exposure to xenobiotics [39]. The concept of conservative transfer of fatty acids through aquatic food webs and their use as biomarkers was first proposed in 1935 by Lovern. Consequently, biomarker analyses of FA have become a crucial tool for elucidating trophic interactions in marine environment [40]. The modification of FA ingested by consumers has significant implications for upper trophic levels, as the value of nutrients available for these levels may be altered or improved. Fatty acids with at least two double bonds in the aliphatic chain (polyunsaturated FAs or PUFAs) typically make up at least a third of all FAs in organisms, where they play several important roles. The lipid profile of bivalve molluscs is typically influenced by their dietary habits and the environmental conditions where they live. The control group samples exhibited a lipid and FA profile comparable to previous studies on mussels from the same harvest site [33], particularly in the predominant SFAs, MUFA, and PUFAs. In any case, following exposure in the laboratory, the mussels exhibited a decline in total lipids and a shift in their SFAs profile. In contrast, exposure to SLS resulted in no changes in MUFAs and a rise in PUFAs, compared to the control group. This observation is consistent with the results of another study by Porretti et al. [33], which also observed a comparable profile in mussels exposed to DEHT, a plasticiser that is widely spread in the environment and identified as a pollutant. So far, no direct studies have investigated the effect of anionic surfactants (such as SLS) on the fatty acid profile of mussels. Nevertheless, there are numerous studies concerning the alteration of the lipid profile of mussels exposed to contaminants such as heavy metals [41,42] and oil [43]. In a study by Albergamo et al. [44], the alteration of fatty acid content in control mussels (from a pristine area) and mussels exposed to different contaminants (an area characterized by the presence of heavy metals and polycyclic aromatic hydrocarbons) was evaluated. The results demonstrated that exposure to these contaminants resulted in a disruption of cellular processes, leading to an alteration of the fatty acid content, particularly in saturated and polyunsaturated acids.

The results of our study show that alpha-linolenic acid (C18:3*ω*-3) was higher in both the BRG-treated group (1.95%) and the mixture (1.99%) than in the control group (1.50%). In all cases, administration of bergamot extracts alone or in combination with SLS (MIX group) resulted in significant restoration of total lipid and FA levels compared to control. Furthermore, there were no significant differences (*p* > 0.05) compared to the control group in the majority of cases. A notable example of this is palmitoleic acid (C16:1 ω-9), which showed a marked increase in the group exposed to the bergamot extract (CTR: 0.10; SLS: 0.04; BRG: 11.07; MIX: 0.13). de Souza et al. [45] posit that palmitoleic acid has anti-inflammatory potential on TNF-α-stimulated endothelial cells, thereby counteracting endothelial dysfunction and affecting atherogenesis and inflammatory processes. Stearidonic acid (C18:4 ω-3), a highly unsaturated PUFA, is metabolised efficiently into eicosapentaenoic acid (EPA) with a bioequivalence of ~5:1, as determined.

An indicator of the risk of various metabolic and cardiovascular diseases is the omega-3 index. In particular, alpha-hydroxy acids (AHAs) are recommended in the so-called “Mediterranean diet”. These include n-3 PUFA, EPA and docosahexaenoic acid (DHA) [46]. However, fish consumption has been shown to be insufficient to meet the body’s needs for these particular fatty acids [47]. In the present study, exposure of mussels to BRG (3.11%) and MIX (3.39%) resulted in a significant increase in this fatty acid.

The exposure of *M. galloprovincialis* to SLS, BRG, and their mixture resulted in a minor decrease in protein metabolism. Mussels in optimal physiological conditions, as observed in the control group, had a higher protein content (20.21%) compared especially to the SLS-treated group (18.16%). The potential effect of contaminants on protein content has not been reported in any study, to date. It should be noted that a similar decrease in protein following the exposure to an organic contaminant, such as DEHT (CTR: 15.95%; DEHT1: 14.76% and DEHT100: 11.46%), was reported in a recent study by Porretti et al. [33].

A study by Vershinin [48] examined the function of carotenoids in various molluscs, including *M. galloprovincialis*. Carotenoids are natural pigments that perform in several biological functions, including protection against oxidation, modulation of the immune system, participation in photosynthesis in photosynthetic molluscs, and the involvement in anaerobic ATP synthesis in cells [49]. Consistent with the findings of Petrunyaka [50], the highest accumulation of carotenoids is observed in organs that accumulate calcium or have high transmembrane calcium transport, and the highest percentage were in hepatopancreas and gills. In addition to which until now discovered, our study showed that exposure to SLS resulted in a significant reduction in carotenoid content, with a mean value of 3.70 mg/kg, compared to the control group (5.99 mg/kg); it can be seen that the mean values were more similar to the untreated group in the BRG-treated group (4.75 mg/kg) and the MIX group (5.77 mg/kg). This highlights how the use of natural extracts, alone or in combination with contaminants, can restore carotenoid levels to physiological levels.

Vitamin E, also known as α-tocopherol, serves as the primary lipid-soluble antioxidant in animals, capable of disrupting free radical chains within cellular membranes [51]. The primary function of tocopherol is, as an antioxidant, protecting membrane structures, essential fatty acids, and vitamin A from oxidation. Filter-feeding molluscs, including bivalves, consume a diet almost exclusively composed of various unicellular marine plants [52], which are the primary source of tocopherols. Mussels are therefore an appropriate subject to investigate tocopherols in shellfish. It has also been shown that vitamin E has an effect on the reproductive processes of animals [53]. Consequently, the vitamin E content of mussels is expected to fluctuate in accordance with the reproductive cycle and across seasonal variations throughout their lifespan. The study by Sigurgisladottir et al. [54] indicates that the mean α-tocopherol content in *M. edulis* from Nova Scotia is greater than that observed in our samples of *M. galloprovincialis*. The reported values for M. edulis range from 3 to 8 μg/g, while those for our *M. galloprovincialis* fall between 0.35 and 0.48 μg/g (*p* > 0.05). These changes are likely attributed to variations in species, geographical origin, and seasonality, and no studies until now have identified alterations in tocopherol content in mussels following an exposure to xenobiotics. Prior studies have already examined the effects of SLS exposure on egg development in mussels [18,19,20]. Based on these findings, it can be concluded that the observed reduction in haemolymph cell viability due to contamination, as indicated by TB staining, is a notable outcome. This can be considered the onset of cellular stress, but simultaneously the onset of the immune response. The resulting data aligns with the hypotheses put forth in the literature, which posit that exposure to surfactants can compromise the functioning of immune cells in aquatic organisms [20]. There were no significant alterations in cell viability observed in DG cells. The percentage of viability of the haemolymph and DG cells obtained in the MIX, which was slightly higher than that observed in the cells of the control group, also suggests a potential protective action of the bergamot peel extract when used in combination with SLS. This result is in accordance with the findings of previous studies which have demonstrated the capacity of natural compounds to alleviate the detrimental effects of environmental pollutants [55,56,57].

Experiments examining the effects on cell viability in mussels caused by exposure to SLS have already been carried out [18,19,20]. Based on these previous results, it can be stated that the observed reduction in the haemolymph viability haemolymph of SLS-exposed cells through the TB staining can be considered as both the beginning of cellular stress and, at the same time, also the beginning of the immune response, which is in line with the hypotheses put forward in the literature that exposure to surfactants can impair the functioning of immune cells in aquatic organisms [37]. The TB analysis easily distinguishes between live and dead cells, confirming that exposure to SLS can result in significant cell mortality. In the present study, the percentage of viable haemolymph in the MIX group was slightly higher than that observed in cells from the control group and SLS exposed, suggesting a potential protective effect of bergamot peel extract when used in combination with SLS. Therefore, the results are consistent with the literature on the ability of natural compounds to mitigate the adverse effects of environmental pollutants [55,56,57].

The RVD test did not give any significant results, but the observed trend provided important information on the osmotic behaviour of DG cells in the mussel under different conditions. The ability to regulate cell volume under physiological conditions is crucial for maintaining homeostasis. The inability of cells exposed to SLS to return to their original shape, together with the reduced ability to swell in both the SLS-treated and MIX groups, suggests an important alteration in this mechanism. Consistent with previous studies, SLS may, therefore, affect the integrity of cell membranes and, thus, the ability of cells to cope with osmotic changes [37]. In contrast, the smaller volumetric reduction observed in the MIX, compared to the group treated with SLS alone, confirms and supports the initial hypothesis of a protective role for bergamot extract against damage caused by the synthetic substances often present in PCPs. Once again, however, the concentration chosen was not sufficient to completely restore the functionality of the cell, demonstrating the need for further tests on this subject.

The upregulation of cytosolic and mitochondrial superoxide dismutase genes (*Cu/ZnSOD* and *MnSOD*) observed following the exposure to SLS, indicates the activation of primary defence mechanisms against oxidative stress [58], aimed at mitigating the harmful effects of SLS. Notably, the combined treatment with SLS and BRG induced a significant decrease in the gene expression of both superoxide dismutase as compared to the SLS group, suggesting the protective role of bergamot extracts in counteracting the adverse effects of this pollutant.

Heat shock proteins (HSPs) are widely recognized for their critical role in stress responses. Remarkably, the observed increase in Hsp70 gene expression in the SLS group (3.9-fold compared to the control) is consistent with a previous study evaluating the cellular responses to PCP in *M. galloprovinjcialis* [37] where exposure to benzisothiazolinone triggered a significant oxidative stress condition associated with Hsp70 upregulation, thereby underscoring the role of Hsp70 in oxidative stress responses. Moreover, the combined treatment with SLS and bergamot extract was able to significantly reduce Hsp70 expression levels as compared to SLS treatment alone. This finding suggests that bergamot extracts may have a role in regulating the expression of heat shock proteins involved in critical cellular processes, such as protein metabolism and the prevention of misfolding and denaturation of cellular components under chronic stress conditions [59]. The CYP4Y1 protein, a member of the CYP4 subfamily, is known to play a key role in the metabolism of various contaminants in *M. galloprovincialis* [60]. In this study, the SLS treatment induced an upregulation of *CYP4Y1* gene expression in gills; a similar trend was previously observed by Impellitteri et al. [61] in mussels exposed to quaternium-15. Notably, the observed modulation of oxidative and cytoprotective related genes strongly suggest the potential beneficial effects of BRG extract.

## 5. Conclusions

In conclusion, this study revealed that exposure to SLS may induce adverse effects in the sentinel species *M. galloprovincialis*, including alterations in key nutritional parameters such as fatty acid profiles, total lipids, total protein, carotenoid and tocopherol content—areas that, to date, have been poorly explored in the scientific literature. Although the results raise concerns about the presence of SLS in Mediterranean waters and its potential bioaccumulation along the food chain, the possible implications for seafood safety and human health must be considered as hypotheses and require further validation. This study also highlights the need for actions to reduce surfactant pollution in aquatic ecosystems and requires a deeper understanding of their impact on marine organisms. Furthermore, the potential of natural nutraceuticals, such as bergamot extract, to mitigate some of the observed alterations has been demonstrated. However, important limitations must be recognised, including the use of a single sentinel species, the absence of long-term exposure studies, and the need to test a wider range of dosages and environmental scenarios. Future research should therefore explore other species, longer exposure periods and investigate possible synergistic effects between SLS and other environmental contaminants.

## Figures and Tables

**Table 1:** Concentration (mg Kg^−1^ ± standard deviation) of bioactive molecules in bergamot peels extract. Each sample was analysed in triplicate.

N°	Compound	Class	Concentration
1	Ferulic acid 4-O-glucoside ^a^	PA	13.36 ± 0.24
2	Sinapoyl glucoside ^b^	PA	169.28 ± 3.20
3	Apigenin 6,8-di-C-β-D-glucoside	F	81.41 ± 1.15
4	Diosmetin-6,8-di-C-glucoside	F	93.05 ± 0.80
5	Eriocitrin	F	409.82 ± 2.72
6	Limonin glucoside	L	24.91 ± 0.34
7	Neoeriocitrin	F	817.89 ± 6.30
8	5-Sinapoyquinic acid ^b^	PA	42.82 ± 0.57
9	Poncirin ^c^	F	1803.86 ± 1.26
10	Diosmetin 8-C-glucoside ^d^	F	166.83 ± 2.39
11	Narirutin	F	1673.57 ± 2.43
12	Naringin	F	658.50 ± 1.40
13	Apigenin 7-O-neohesperidoside ^c^	F	31.52 ± 0.92
14	Deacetyl nomilin glucoside ^e^	L	806.94 ± 0.44
15	Neodiosmin ^c^	F	1187.27 ± 2.26
16	Apigenin 7-O-neohesperidoside-4-glucoside ^c^	F	6.80 ± 0.11
17	Neohesperidin	F	12,907.64 ± 5.52
18	Nomilin glucoside	F	905.32 ± 2.16
19	Nomilinic acid glucoside	F	466.10 ± 0.64
20	Apigenin 7-O-diglucuronide ^c^	F	19.87 ± 0.27
21	Melitidin	F	298.54 ± 0.27
22	Brutieridin	F	1029.44 ± 5.25
23	Ichangin ^f^	L	34.70 ± 0.05
24	Obacunoic acid ^f^	L	32.02 ± 0.01
25	Limonin	L	79.04 ± 0.17
26	Nomilin	L	124.49 ± 0.20
	*All*		*23,884.84* ± 6.84

Bioactive molecules were quantitatively determined based on calibration curves obtained with the correspondent standard compound: ^a^ ferulic acid; ^b^ synapic acid; ^c^ apigenin 6,8-di-C-glucoside; ^d^ diosmetin 6,8-di-C-glucoside; ^e^ nomilin glucoside; ^f^ limonin.

**Table 2:** Number of byssal plaques and length of plaque of *M. galloprovincialis* detected by stereomicroscope in the control and treatment groups (SLS, BRG, MIX). Data are presented as mean ± SD of duplicate analyses.

	Control	SLS (0.010 mg/L)	BRG (5 mg/L)	MIX (0.010 mg/L SLS + 5 mg/L BRG)
Number of byssal plaque	102.75 ± 14.931 ^a^	60.75 ± 19.312 ^a^	101.75 ± 30.184 ^a,b^	38.00 ± 29.472 ^a,c^
Length of plaque (mm)	0.76 ± 0.142 ^a^	0.61 ± 0.125 ^b^	0.61 ± 0.111 ^b,c^	0.65 ± 0.141 ^b,c,d^

a–d: different superscript letters in the same row indicate significantly different values for a given parameter (*p* < 0.05 by post hoc Tukey’s HSD test); same superscript letters in the same row indicate not significantly different values for a given parameter (*p* > 0.05 by post hoc Tukey’s HSD test).

**Table 3:** Total lipid (%, fw) and FA profile (% of total fatty acids, fw) of control, SLS, BRG and MIX treated pools of *M. galloprovincialis*. Data are represented as mean ± SD of triplicate analysis.

Analyte (%)	Tested Groups			
	Control	SLS (0.010 mg/L)	BRG (5 mg/L)	MIX (0.010 mg/L SLS + 5 mg/L BRG)
*Total lipid*	2.21 ± 0.59 ^a^	1.43 ± 0.22 ^a^	2.06 ± 0.17 ^a^	1.93 ± 0.18 ^a^
C14:0	3.33 ± 0.44 ^a^	1.03 ±0.03 ^b^	3.60 ± 0.15 ^a^	3.96 ± 0.14 ^a^
C15:0	0.63 ± 0.06 ^a^	0.46 ± 0.10 ^a^	0.62 ± 0.10 ^a^	0.82 ± 0.11 ^a^
C16:0	19.01 ± 0.13 ^a^	15.97 ± 0.27 ^b^	18.92 ± 0.37 ^a^	20.02 ± 0.65 ^a^
C17:0	1.25 ± 0.06 ^a^	1.61 ± 0.11 ^b^	1.25 ± 0.08 ^a^	1.11 ± 0.60 ^a^
C18:0	6.63 ± 0.73 ^a^	8.77 ±0.27 ^b^	6.20 ± 0.14 ^a^	5.15 ± 0.13 ^a^
C20:0	1.67 ± 0.11 ^a^	1.52 ± 0.15 ^a,b^	1.03 ± 0.06 ^b^	1.41 ± 0.14 ^a,b^
*Σ SFA*	*32.80 ± 0.44 ^a^*	*29.67 ±0.24 ^b^*	*31.88 ± 0.49 ^a^*	*32.68 ± 0.30 ^a^*
C 14:1	0.64 ± 0.1 ^a^	0.52 ± 0.15 ^a^	0.55 ± 0.17 ^a^	0.76 ± 0.06 ^a^
C 15:1	0.07 ± 0.01 ^a^	0.08 ± 0.02 ^a^	0.11 ± 0.01 ^a^	0.05 ± 0.01 ^a^
C16:1 ω-9	0.10 ± 0.03 ^a^	0.04 ± 0.01 ^a^	11.07 ± 0.23 ^b^	0.13 ± 0.02 ^a^
C16:1 ω-7	6.17 ± 0.21 ^a^	2.88 ± 0.08 ^b^	0.16 ± 0.07 ^c^	8.64 ± 0.31 ^d^
C17:1	0.30 ± 0.03 ^a^	0.30 ± 0.04 ^a^	0.30 ± 0.03 ^a^	0.35 ± 0.07 ^a^
C18:1 ω-9	3.50 ± 0.15 ^a^	4.1 ± 0.18 ^b^	2.74 ± 0.13 ^b^	1.84 ± 0.08 ^b^
C18:1 ω-7	2.64 ±0.13 ^a^	2.52 ± 0.07 ^a^	3.54 ± 0.27 ^b^	3.04 ± 0.10 ^a,b^
C20:1 ω-11	2.49 ± 0.14 ^a^	3.06 ± 0.07 ^b^	1.57 ± 0.08 ^a,c^	2.20 ± 0.13 ^b^
C20:1 ω-7	1.96 ± 0.12 ^a^	2.08 ± 0.10 ^a^	2.06 ± 0.04 ^a^	2.01 ± 0.03 ^a^
C20:1 ω-9	0.61 ± 0.11 ^a^	3.29 ± 0.15 ^b^	2.54 ± 0.38 ^b^	2.74 ± 0.34 ^b^
C22:1 ω-11	0.20 ± 0.03 ^a^	0.43 ± 0.13 ^b^	0.19 ± 0.03 ^a^	0.08 ± 0.01 ^b^
C22:1 ω-9	0.61 ± 0.07 ^a^	0.91 ± 0.17 ^a,c^	0.33 ± 0.03 ^a,b^	0.13 ± 0.04 ^b^
*Σ MUFA*	*19.33 ± 0.34 ^a^*	*20.29 ± 0.35 ^a^*	*23.14 ± 0.15 ^b^*	*22.07 ± 0.65 ^b^*
C18:2 ω-6	0.96 ± 0.07 ^a^	0.64 ± 0.13 ^a,b^	1.01 ± 0.02 ^a,c^	1.19 ± 0.05 ^a,c^
C18:3 ω-6	0.04 ± 0.01 ^a^	0.05 ± 0.01 ^a^	0.02 ± 0.01 ^a^	0.04 ± 0.01 ^a^
C18:3 ω-3	1.50 ± 0.40 ^a^	0.99 ± 0.04 ^a,b^	1.95 ± 0.11 ^a,c^	1.99 ± 0.06 ^a,c^
C18:4 ω-3	2.76 ± 0.27 ^a^	1.04 ± 0.04 ^b^	3.11 ± 0.04 ^a,c^	3.39 ± 0.09 ^c^
C18:4 ω-1	0.10 ± 0.01 ^a^	0.14 ± 0.03 ^a^	0.09 ± 0.03 ^a^	0.11 ± 0.01 ^a^
C20:2 ω-6	0.25 ± 0.10 ^a^	0.30 ± 0.05 ^a^	0.18 ± 0.04 ^a^	0.21 ± 0.03 ^a^
C20:3 ω-6	0.09 ± 0.01 ^a^	0.15 ± 0.06 ^a^	0.08 ± 0.01 ^a^	0.09 ± 0.03 ^a^
C20:3 ω-3	0.14 ± 0.03 ^a^	0.11 ± 0.06 ^a^	0.08 ± 0.01 ^a^	0.09 ± 0.04 ^a^
C20:4 ω-6	3.50 ± 0.41 ^a^	4.85 ± 0.24 ^b^	3.01 ± 0.06 ^a^	3.08 ± 0.11 ^a^
C20:4 ω-3	0.23 ± 0.13 ^a^	0.28 ± 0.08 ^a^	0.13 ± 0.03 ^a,b^	0.53 ± 0.08 ^a,c^
C20:5 ω-3	20.25 ± 0.86 ^a^	20.96 ± 0.23 ^a^	20.30 ± 0.31 ^a^	20.82 ± 1.03 ^a^
C21:5 ω-3	0.85 ± 0.10 ^a^	0.83 ± 0.11 ^a^	0.55 ± 0.07 ^a^	0.59 ± 0.07 ^a^
C22:2	0.07 ± 0.01 ^a^	0.06 ± 0.01 ^a^	0.03 ± 0.01 ^a^	0.03 ± 0.01 ^a^
C22:4 ω-6	0.37 ± 0.04 ^a^	0.57 ± 0.06 ^a,b^	0.32 ± 0.07 ^a^	0.30 ± 0.07 ^a^
C22:5 ω-6	0.47 ± 0.03 ^a^	0.66 ± 0.04 ^a,b^	0.35 ± 0.06 ^a^	0.35 ± 0.08 ^a^
C22:5 ω-3	0.67 ± 0.06 ^a^	0.78 ± 0.10 ^a^	0.30 ± 0.03 ^b^	0.71 ± 0.10 ^a^
C22:6 ω-3	13.70 ± 0.88 ^a^	17.35 ± 0.75 ^b^	11.51 ± 0.41 ^a^	11.73 ± 0.30 ^a^
*Σ PUFA*	*45.06 ± 0.31 ^a^*	*49.76 ± 0.42 ^b^*	*42.96 ± 0.44 ^a^*	*45.25 ± 0.35 ^a^*
Σ ω3	39.25 ± 0.39 ^a^	42.34 ± 0.56 ^b^	37.87 ± 0.68 ^a^	39.85 ± 0.57 ^a,b^
Σ ω6	5.31 ± 0.35 ^a^	6.65 ± 0.04 ^b^	4.65 ± 0.11 ^a^	4.96 ± 0.10 ^a^
ω6/ω3	0.14 ± 0.09 ^a^	0.16 ± 0.02 ^a^	0.12 ± 0.01 ^a^	0.12 ± 0.02 ^a^

a–d: different superscript letters in the same row indicate significantly different values for a given parameter (*p* < 0.05 by post hoc Tukey’s HSD test); same superscript letters in the same row indicate not significantly different values for a given parameter (*p* > 0.05 by post hoc Tukey’s HSD test).

**Table 4:** Total protein (%, fw), Carotenoids (β-carotene, ppm), Tocopherols (Vitamin E, ppm) of control and treated pools (i.e., SLS, BRG, MIX) of *M. galloprovincialis*. Data are presented as mean ± SD of triplicate analysis.

	Test Groups			
	Control	SLS (0.010 mg/L)	BRG (5 mg/L)	MIX (0.010 mg/L SLS + 5 mg/L BRG)
**Total protein (%)**	20.212 ± 0.603 ^a^	18.164 ± 0.312 ^a^	18.415 ± 0.494 ^a^	18.567 ± 0.624 ^a^
**Carotenoids** **(β-carotene, mg/Kg)**	5.991 ± 0.963 ^a^	3.698 ± 0.163 ^ab^	4.754 ± 0.832 ^a^	7.444 ± 0.452 ^ac^
**Tocopherols** **(Vitamin E, mg/Kg)**	0.459 ± 0.101 ^a^	0.402 ± 0.061 ^a^	0.353 ± 0.080 ^a^	0.481± 0.103 ^a^

a–c: different superscript letters in the same row indicate significantly different values for a given parameter (*p* < 0.05 by post hoc Tukey’s HSD test); same superscript letters in the same row indicate not significantly different values for a given parameter (*p* > 0.05 by post hoc Tukey’s HSD test).

**Table 5:** Cell viability tests of *M. galloprovincialis* haemocytes and digestive gland cells from control and treated groups (i.e., SLS, BRG, MIX). Data are presented as mean ± SD of triplicate analyses.

	Test Groups			
	Control	SLS (0.010 mg/L)	BRG (5 mg/L)	MIX (0.010 mg/L SLS + 5 mg/L BRG)
**Haemolymph cells (TB, %)**	98.41 ± 1.65 ^a^	93.54 ± 2.46 ^b^	97.22 ± 2.62 ^a,b,c^	96.00 ± 2.98 ^a,b,d^
**Digestive glands cells (TB, %)**	98.53 ± 0.85 ^a^	97.83 ± 1.55 ^a^	98.81 ± 0.79 ^a^	98.72 ± 0.82 ^a^

a–d: different superscript letters in the same row indicate significantly different values for a given parameter (*p* < 0.05 by post hoc Tukey’s HSD test); same superscript letters in the same row indicate not significantly different values for a given parameter (*p* > 0.05 by post hoc Tukey’s HSD test).

## Data Availability

Data will be made available under request.

## References

[1] Colitti M., Stefanon B., Gabai G., Gelain M.E., Bonsembiante F. (2019). Oxidative stress and nutraceuticals in the modulation of the immune function: Current knowledge in animals of veterinary interest. Antioxidants.

[2] Hassan S., Meenatchi R., Pachillu K., Bansal S., Brindangnanam P., Arockiaraj J., Kiran G.S., Selvin J. (2022). Identification and characterization of the novel bioactive compounds from microalgae and cyanobacteria for pharmaceutical and nutraceutical applications. J. Basic. Microbiol..

[3] Kumar K., Kumar S. (2015). Role of nutraceuticals in health and disease prevention: A review. South Asian J. Food Technol. Environ..

[4] Varghese T., SanalEbeneeza S.A.D., Pal A.K. (2021). Mitigation of stress in fish through nutraceuticals. Development.

[5] Gomes I.B., Maillard J.Y., Simões L.C., Simões M. (2020). Emerging contaminants affect the microbiome of water systems—Strategies for their mitigation. NPJ Clean. Water.

[6] Maiuolo J., Carresi C., Gliozzi M., Musolino V., Scarano F., Coppoletta A.R., Guarnieri L., Nucera S., Scicchitano M., Bosco F. (2021). Effects of bergamot polyphenols on mitochondrial dysfunction and sarcoplasmic reticulum stress in diabetic cardiomyopathy. Nutrients.

[7] Costa R., Dugo P., Navarra M., Raymo V., Dugo G., Mondello L. (2010). Study on the chemical composition variability of some processed bergamot (*Citrus bergamia*) essential oils. Flav. Frag. J..

[8] Salerno R., Casale F., Calandruccio C., Procopio A. (2016). Characterization of flavonoids in *Citrus bergamia* (Bergamot) polyphenolic fraction by liquid chromatography–high resolution mass spectrometry (LC/HRMS). Pharma. Nutr..

[9] Navarra M., Mannucci C., Delbò M., Calapai G. (2015). *Citrus bergamia* essential oil: From basic research to clinical application. Front. Pharm..

[10] Russo M., Bonaccorsi I., Inferrera V., Dugo P., Mondello L. (2015). Underestimated sources of flavonoids, limonoids and dietary fiber: Availability in orange’s by-products. J. Funct. Foods.

[11] Musolino V., Gliozzi M., Nucera S., Carresi C., Maiuolo J., Mollace R., Mollace V. (2019). The effect of bergamot polyphenolic fraction on lipid transfer protein system and vascular oxidative stress in a rat model of hyperlipemia. Lip. Health Dis..

[12] Kumar S., Talwar S., Krishnan S., Kaur P., Dhir A. (2021). Purchasing natural personal care products in the era of fake news? The moderation effect of brand trust. J. Retail. Consum. Serv..

[13] Marteinson S.C., Lawrence M.J., Taranu Z.E., Kosziwka K., Taylor J.J., Green A., Winegardner A.K., Rytwinski T., Reid J., Dubetz C. (2022). Increased use of sanitizers and disinfectants during the COVID-19 pandemic: Identification of antimicrobial chemicals and considerations for aquatic environmental contamination. Eniviron. Rev..

[14] Morin-Crini N., Lichtfouse E., Liu G., Balaram V., Ribeiro A.R.L., Lu Z., Stock F., Carmona E., Teixeira M.R., Picos-Corrales L.A. (2022). Worldwide cases of water pollution by emerging contaminants: A review. Environ. Chem. Lett..

[15] Chirani M.R., Kowsari E., Teymourian T., Ramakrishna S. (2021). Environmental impact of increased soap consumption during COVID-19 pandemic: Biodegradable soap production and sustainable packaging. Sci. Total Environ..

[16] Freitas R., Coppola F., Meucci V., Battaglia F., Soares A.M., Pretti C., Faggio C. (2021). The influence of salinity on sodium lauryl sulfate toxicity in *Mytilus galloprovincialis*. Environ. Toxicol. Pharm..

[17] Cserháti T., Forgács E., Oros G. (2002). Biological activity and environmental impact of anionic surfactants. Environ. Int..

[18] Freitas R., Arrigo F., Coppola F., Meucci V., Battaglia F., Soares A.M., Pretti C., Faggio C. (2023). Combined effects of temperature rise and sodium lauryl sulfate in the Mediterranean mussel. Environ. Toxicol. Pharm..

[19] Lopes J., Coppola F., Russo T., Maselli V., Di Cosmo A., Meucci V., Soares A.M.V.M., Pretti C., Polese G., Freitas R. (2022). Behavioral, physiological and biochemical responses and differential gene expression in *Mytilus galloprovincialis* exposed to 17 alpha-ethinylestradiol and sodium lauryl sulfate. J. Hazard. Mater..

[20] Paciello S., Russo T., De Marchi L., Soares A.M., Meucci V., Pretti C., Pretti C., He Y., Della Torre C., Freitas R. (2023). Sub-lethal effects induced in *Mytilus galloprovincialis* after short-term exposure to sodium lauryl sulfate: Comparison of the biological responses given by mussels under two temperature scenarios. Comp. Biochem. Physiol. C Toxicol. Pharmacol..

[21] Beyer J., Green N.W., Brooks S., Allan I.J., Ruus A., Gomes T., Bråte I.L., Schøyen M. (2017). Blue mussels (*Mytilus edulis* spp.) as sentinel organisms in coastal pollution monitoring: A review. Mar. Environ. Res..

[22] Kournoutou G.G., Giannopoulou P.C., Sazakli E., Leotsinidis M., Kalpaxis D.L., Dinos G.P. (2020). Oxidative damage of mussels living in seawater enriched with trace metals, from the viewpoint of proteins expression and modification. Toxics.

[23] Miglioli A., Tredez M., Boosten M., Sant C., Carvalho J.E., Dru P., Canesi L., Schubert M., Dumollard R. (2024). The Mediterranean mussel *Mytilus galloprovincialis*: A novel model for developmental studies in mollusks. Development.

[24] De Troch M., Boeckx P., Cnudde C., Van Gansbeke D., Vanreusel A., Vincx M., Caramujo M.J. (2012). Bioconversion of fatty acids at the basis of marine food webs: Insights from a compound-specific stable isotope analysis. Mar. Ecol. Prog. Ser..

[25] Kelly J.R., Scheibling R.E. (2012). Fatty acids as dietary tracers in benthic food webs. Mar. Ecol. Prog. Ser..

[26] Chandan N.K., Kumari R., Siddaiah G.M. (2020). Role of nutraceuticals in fish feed. Fish Nutrition and Its Relevance to Human Health.

[27] Naseemashahul S., Sahu N.P., Sardar P., Fawole F.J. (2021). Effects of nutraceutical conglomerate on growth and antioxidant status of *Labeo rohita* fingerlings. AFST.

[28] Naserabad S.S., Zarei S., Rahimi J., Ghafouri Z., Mouludi-Saleh A., Banaee M. (2024). Protective effects of *Allium jesdianum* essential oil on rainbow trout (*Oncorhynchus mykiss*) exposed to sub-lethal toxicity of cypermethrin. Aquat. Toxicol..

[29] Russo M., Bonaccorsi I., Torre G., Sarò M., Dugo P., Mondello L. (2014). Underestimated sources of flavonoids, limonoids and dietary fiber: Availability in lemon’s by-products. J. Funct. Foods.

[30] Russo M., Dugo P., Marzocco S., Inferrera V., Mondello L. (2016). Multidimensional preparative liquid chromatography to isolate flavonoids from bergamot juice and evaluation of their anti-inflammatory potential. J. Separat. Sci..

[31] Russo M., Arigò A., Calabrò M.L., Farnetti S., Mondello L., Dugo P. (2016). Bergamot (*Citrus bergamia* Risso) as a source of nutraceuticals: Limonoids and flavonoids. J. Funct. Foods.

[32] Bolognesi C., Fenech M. (2012). Mussel micronucleus cytome assay. Nat. Protoc..

[33] Porretti M., Impellitteri F., Caferro A., Albergamo A., Litrenta F., Filice M., Faggio C. (2023). Assessment of the effects of non-phthalate plasticizer DEHT on the bivalve molluscs *Mytilus galloprovincialis*. Chemosphere.

[34] Rotondo A., La Torre G.L., Bartolomeo G., Rando R., Vadalà R., Zimbaro V., Salvo A. (2021). Profile of carotenoids and tocopherols for the characterization of lipophilic antioxidants in “ragusano” cheese. Appl. Sci..

[35] Lo Turco V., Litrenta F., Nava V., Albergamo A., Rando R., Bartolomeo G., Potortì A.G., Di Bella G. (2023). Effect of Filtration Process on Oxidative Stability and Minor Compounds of the Cold-Pressed Hempseed Oil during Storage. Antioxidants.

[36] Impellitteri F., Riolo K., Zicarelli G., Porretti M., Multisanti C.R., Piccione G., Giannetto A., Faggio C. (2025). Evaluation of cellular and physiological alterations of cells from *Mytilus galloprovincialis* exposed to benzisothiazolinone. Ecotoxicol. Environ. Saf..

[37] Riolo K., Franco G.A., Marino Y., Ferreri A., Oliva S., Parrino V., Savastano D., Cuzzocrea S., Gugliandolo E., Giannetto A. (2025). Protein hydrolysates from *Hermetia illucens* trigger cellular responses to cope with LPS-induced inflammation and oxidative stress in L-929 cells. Anim. Cells Syst..

[38] Bondi C.A., Marks J.L., Wroblewski L.B., Raatikainen H.S., Lenox S.R., Gebhardt K.E. (2015). Human and environmental toxicity of sodium lauryl sulfate (SLS): Evidence for safe use in household cleaning products. Environ. Health Insights.

[39] Asio J.R.G., Garcia J.S., Antonatos C., Sevilla-Nastor J.B., Trinidad L.C. (2023). Sodium lauryl sulfate and its potential impacts on organisms and the environment: A thematic analysis. Emerg. Contamin.

[40] Maazouzi C., Masson G., Izquierdo M.S., Pihan J.C. (2008). Chronic copper exposure and fatty acid composition of the amphipod Dikerogammarus villosus: Results from a field study. Environ. Poll..

[41] Filimonova V., Goncalves F., Marques J.C., De Troch M., Goncalves A.M. (2016). Fatty acid profiling as bioindicator of chemical stress in marine organisms: A review. Ecol. Indic..

[42] Dalsgaard J., John M.S., Kattner G., Müller-Navarra D., Hagen W. (2003). Fatty acid trophic markers in the pelagic marine environment. Adv. Mar. Bio.

[43] Fokina N.N., Ruokolainen T.R., Nemova N.N., Bakhmet I.N. (2013). Changes of blue mussels *Mytilus edulis* L. lipid composition under cadmium and copper toxic effect. Biol. Trace Elem. Res..

[44] Albergamo A., Rigano F., Purcaro G., Mauceri A., Fasulo S., Mondello L. (2016). Free fatty acid profiling of marine sentinels by nanoLC-EI-MS for the assessment of environmental pollution effects. Sci. Total Environ..

[45] de Souza C.O., Valenzuela C.A., Baker E.J., Miles E.A., Rosa Neto J.C., Calder P.C. (2018). Palmitoleic acid has stronger anti-inflammatory potential in human endothelial cells compared to oleic and palmitic acids. Mol. Nutr. Food Res..

[46] Finicelli M., Di Salle A., Galderisi U., Peluso G. (2022). The Mediterranean diet: An update of the clinical trials. Nutrients.

[47] Whelan J., Gouffon J., Zhao Y. (2012). Effects of dietary stearidonic acid on biomarkers of lipid Metabolism4. J. Nutr..

[48] Vershinin A. (1996). Carotenoids in mollusca: Approaching the functions. Comp. Biochem. Physiol. B.

[49] Nagy I.Z. (1977). Cytosomes (yellow pigment granules) of molluscs as cell organelles of anoxic energy production. International Review of Cytology.

[50] Petrunyaka V.V. (1982). Localization and role of carotenoids in molluscan neurons. Cell. Mol. Neurobiol..

[51] Packer L., Landvik S. (1989). Vitamin E: Introduction to biochemistry and health benefits. Ann. N. Y. Acad. Sci..

[52] Page H.M., Ricard Y.O. (1990). Food availability as a limiting factor to mussel *Mytilus edulis* growth in California coastal waters. Fish. Bull..

[53] Nelson J.S. (1981). Pathology of Vitamin E Deficiency. https://www.cabidigitallibrary.org/doi/full/10.5555/19801411774.

[54] Sigurgisladottir S., Ackman R.G., O’keefe S.F. (1993). Selective deposition of α-tocopherol in lipids of farmed blue mussels (*Mytilus Edulis*). J. Food Lip..

[55] Omidkhoda S.F., Razavi B.M., Hosseinzadeh H. (2019). Protective effects of *Ginkgo biloba* L. against natural toxins, chemical toxicities, and radiation: A comprehensive review. Phytother. Res..

[56] Rathod N.B., Ranveer R.C., Benjakul S., Kim S.K., Pagarkar A.U., Patange S., Ozogul F. (2021). Recent developments of natural antimicrobials and antioxidants on fish and fishery food products. Compr. Rev. Food Sci. Food Saf..

[57] Smital T., Luckenbach T., Sauerborn R., Hamdoun A.M., Vega R.L., Epel D. (2004). Emerging contaminants—Pesticides, PPCPs, microbial degradation products and natural substances as inhibitors of multixenobiotic defense in aquatic organisms. Mutat. Res. Fundam. Mol. Mech. Mutagen.

[58] Wang Q., Yuan Z., Wu H., Liu F., Zhao J. (2013). Molecular characterization of a manganese superoxide dismutase and copper/zinc superoxide dismutase from the mussel *Mytilus galloprovincialis*. Fish. Shell. Imm.

[59] Freeman B.C., Morimoto R.I. (1996). The human cytosolic molecular chaperones hsp90, hsp70 (hsc70) and hdj-1 have distinct roles in recognition of a non-native protein and protein refolding. EMBO J..

[60] D’Agata A., Cappello T., Maisano M., Parrino V., Giannetto A., Brundo M.V., Mauceri A. (2014). Cellular biomarkers in the mussel *Mytilus galloprovincialis* (Bivalvia: Mytilidae) from Lake Faro (Sicily, Italy). Ital. J. Zool..

[61] Impellitteri F., Riolo K., Multisanti C.R., Zicarelli G., Piccione G., Faggio C., Giannetto A. (2024). Evaluating quaternium-15 effects on *Mytilus galloprovincialis*: New insights on physiological and cellular responses. Sci. Total Environ..

